# α-cell SLC38A5 supports amino acid-induced α-cell proliferation and glucagon secretion

**DOI:** 10.3389/fendo.2026.1830329

**Published:** 2026-06-15

**Authors:** Katelyn Sellick, Anna Marie R. Schornack, Tyler J. Rodgers, Fedora O. Ogodo, Mounika Aramandla, Jade E. Stanley, Walter Siv, Matthew Shou, Nitin C. Shankar, Soham S. Saraf, Diana E. Stanescu, E. Danielle Dean

**Affiliations:** 1 Division of Diabetes, Endocrinology, and Metabolism, Vanderbilt University Medical Center, Nashville, TN, United States; 2 Department of Molecular Physiology and Biophysics, Vanderbilt University, Nashville, TN, United States; 3 Division of Endocrinology and Diabetes, The Children’s Hospital of Philadelphia, Philadelphia, PA, United States; 4 Department of Pediatrics, Perelman School of Medicine, University of Pennsylvania, Philadelphia, PA, United States

**Keywords:** alpha cell proliferation, amino acid transport, glucagon, mTOR, nutrient sensing, pancreatic islets, SLC38A5

## Abstract

**Background:**

Pancreatic α cells are key regulators of glucose homeostasis, and dysregulated glucagon secretion contributes to hyperglycemia in diabetes. Amino acids strongly stimulate α-cell proliferation and glucagon release, yet the transport mechanisms underlying these responses remain incompletely defined. The neutral amino acid transporter SLC38A5 is highly enriched in α cells, but its α-cell–autonomous role in nutrient sensing is unclear.

**Methods:**

We generated an α-cell-specific *Slc38a5* knockout mouse model to examine the direct contribution of SLC38A5 to α-cell proliferation and nutrient-sensitive signaling. Mice were treated with a monoclonal GCGR antibody (GCGR mAb) to induce hyperaminoacidemia. α-cell proliferation and ribosomal protein S6 phosphorylation were assessed under conditions of elevated circulating amino acids.

**Results:**

*Slc38a5/SLC38A5* gene expression was highly enriched in pancreatic α cells in both mice and humans. Despite comparable increases in circulating amino acids upon GCGR mAb treatment, α-cell-specific deletion of *Slc38a5* markedly attenuated amino acid-induced glucagon secretion and α-cell proliferation in both sexes demonstrating an α-cell-autonomous requirement for SLC38A5. Phosphorylation of ribosomal protein S6 at Ser235/236 or Ser240/244 was unaffected by SLC38A5 deletion, indicating that global nutrient-responsive signaling at this site is largely SLC38A5-independent.

**Conclusion:**

These data identify SLC38A5 as a mediator linking amino acid availability to α-cell proliferation, highlighting its role in α-cell nutrient sensing and adaptation.

## 1 Introduction

Pancreatic islet hormone secretion is tightly regulated by nutrient availability to maintain systemic glucose homeostasis (1, 2). While β cells have historically dominated the focus of metabolic research, α cells and their hormone glucagon are now recognized as key drivers of dysregulated glycemia in diabetes (3–5). Inappropriately elevated glucagon secretion contributes to fasting and postprandial hyperglycemia, underscoring the importance of understanding the metabolic and nutrient-sensing mechanisms that regulate α-cells (6–11).

Glucagon signaling in liver stimulates hepatic glucose output via glycogenolysis and gluconeogenesis with concomitant ureagenesis (11). Loss of glucagon signaling leads to improved glycemia in animal models (12) and in clinical trials for both individuals with either type 1 diabetes or type 2 diabetes (13). However, loss of glucagon signaling results in profound hyperaminoacidemia, hyperglucagonemia, and α-cell hyperplasia (14). Thus, liver glucagon signaling is a critical regulator of both glycemia and circulating amino acid levels. Among nutrient cues, amino acids are particularly potent regulators of α cells (15, 16). Circulating amino acids in turn stimulate glucagon secretion and promote α-cell proliferation, positioning α cells as central sensors of amino acid availability (17–19). Together, these studies demonstrate a potent endocrine feedback loop termed the liver- α-cell axis (14, 17, 20). These effects require coordinated amino acid transport and metabolism in both liver and α-cells (14, 21). However, the specific amino acid transporters that govern amino acid uptake in α cells and their roles in α-cell function are not yet fully understood.

Amino acid transporters are central regulators of pancreatic α-cell biology, linking nutrient availability to glucagon secretion, cellular metabolism, and proliferation (15). α cells are uniquely sensitive to circulating amino acids, relying on transport systems such as SLC38A5, SLC38A4, SLC7A2, SLC3A2 and SLC7A5 to mediate uptake of neutral and essential amino acids (19, 22). These transporters support glucagon release both directly by providing substrates that stimulate secretion and indirectly through metabolic coupling and signaling pathways (21). The sodium-coupled neutral amino acid transporter SLC38A5 has emerged as a key regulator of α-cell biology (14, 23). SLC38A5 belongs to the system N family of amino acid transporters and mediates sodium-dependent uptake of neutral amino acids, including glutamine, asparagine, and histidine (24). Previous research has demonstrated that *Slc38a5* is highly enriched in pancreatic α cells and is required for α cell hyperplasia induced by glucagon receptor (GCGR) inhibition in mice (23). However, previous studies relied on systemic manipulations that do not distinguish direct effects in α cells from indirect effects elsewhere. The role of how α-cell-specific SLC38A5 contributes to proliferation and mTORC1 signaling is still unclear. Additionally, potential sex-specific differences and the relationship between circulating amino acids and α-cell proliferation have not been systematically examined.

In this study, we created a novel α cell-specific knockout of *Slc38a5* to examine the selective effects of SLC38A5 on glucagon secretion, α cell proliferation, and mTORC1 signaling. We also evaluated correlations between circulating amino acids and α-cell proliferation and compared outcomes between male and female mice. By focusing specifically on α cells, this approach lets us define the direct contribution of SLC38A5 to α cell growth and function and provides new insight into how amino acid availability influences α cell adaptations *in vivo*.

## 2 Materials and methods

### 2.1 Generation of alpha cell specific knockouts of Slc38a5

All mouse studies were performed at Vanderbilt University Medical Center and approved by the Institutional Animal Care and Use Committee. Mice were housed on a 12:12 hour light:dark cycle with ad libtum access to standard rodent chow, unless indicated for fasting purposes, and water. To generate α cell–specific knockouts of *Slc38a5*, *Slc38a5*^flox/flox^ mice were crossed with tamoxifen-inducible Gcg-Cre mice (B6;129S4-Gcg^em1(cre/ERT2)Khk^/Mmjax; Jackson Laboratories MMRRC strain #042277-JAX). Offspring carrying both the floxed *Slc38a5* allele and the *Cre* recombinase transgene were used as α cell-specific *Slc38a5* knockout mice, while littermates lacking *Cre* served as controls. These mice were generated on a mixed background.

Cre recombination was induced at 6 weeks of age using tamoxifen (Sigma, T5648), which was dissolved in corn oil (Sigma, C8267) at a concentration of 20 mg/mL by rotating overnight at room temperature. Mice received intraperitoneal injections of tamoxifen (100 µL per dose) once daily for three consecutive days. A two-week washout period was allowed prior to initiation of experimental procedures to ensure complete recombination and clearance of tamoxifen.

### 2.2 GCGR monoclonal antibody treatment and metabolic assessment

Mice were treated with either a glucagon receptor (GCGR) monoclonal antibody (Eli Lilly), an isotype-matched IgG control antibody (Eli Lilly), or sterile Dulbecco’s phosphate-buffered saline (DPBS; Corning, MT21031CV). Antibodies were administered via intraperitoneal injection at a dose of 10 mg/kg (10 µg/g body weight) in a total volume of 100 µL. Injections were performed once weekly for either two or six weeks, as indicated.

Body weight and fed blood glucose levels were measured weekly prior to injections. Blood glucose measurements were obtained from a small drop of blood collected via tail nick using a handheld glucometer.

### 2.3 Immunofluorescence and image analysis

After treatment periods, pancreata were collected. Histology samples were fixed in 4% paraformaldehyde, embedded in OCT (Fisher Scientific) and stored at −80 °C until use.

Whole mouse pancreas was sectioned on a cryostat at a thickness of 8μm per section. The full depth of the pancreas was sectioned by seven repeated steps of sectioning away 150μm and collecting 10 sections onto slides. In this way 7 depths of 150μm were collected from each pancreas. For islet cell mass analysis, one section from each of the seven depths was stained for C-peptide (Invitrogen, PA-85595) to mark β cells, glucagon (LSBio, LS-C202759) to mark α cells and somatostatin (Santa Cruz, sc-7819) to mark δ cells. Whole pancreatic sections were imaged on Leica DMi8 Inverted Microscope (LasX) and islet cell areas were analyzed using ImageJ (Fiji). Total pancreatic islet cell masses were calculated as described previously (Golson 2014). Briefly, islet cell areas from each of seven depths were normalized to total pancreas section area, areas from each of the seven sections were summed and the sum multiplied by total pancreas mass to achieve an estimate of the percent of total mass.

For α cell proliferation analysis, pancreas form mice treated for two weeks with GCGR mAb were sectioned and immunostained for the proliferation marker, Ki67 (Abcam, ab15580), and glucagon to mark α cells. Whole sections were imaged on the Leiva DMi8 Inverted Microscope and analyzed on ImageJ software (Fiji). Percent α cell proliferation was calculated from at least 1000 α cells per animal by dividing Ki76^+^/glucagon^+^ cells by total glucagon^+^ cells. Similarly, percent Slc38a5^+^ α cells were calculated by staining for Slc38a5 (Santa Cruz, sc-50682) and glucagon, imaging and analyzing as above. Amino acid-stimulated α cell proliferation has been shown to be mTOR-dependent. We verified this mTOR-dependence by staining for a target of mTOR1 activity, phosphorylation of ribosomal protein S6 (Cell Signaling: pS6^235/236^, 4858S; pS6^240/244^, 5364S) and glucagon. Percent of positive cells was calculated in the same manner as α cell proliferation.

### 2.4 RNA sequencing analysis

Mouse single-cell RNA sequencing data sets are publicly available at https://tabula-muris.sf.czbiohub.org/visualizations. The facs sorted pancreas data set was utilized to visualize the mean expression of *Slc38a5* in pancreas cell types.

Human single cell RNA sequencing data sets are publicly available at https://doi.org/10.5281/zenodo.15596314. Single-cell RNA sequencing (scRNA-seq) data was obtained from the PanKBase dataset (version 3.3) and loaded into R (v4.2) using the Seurat package (v4.3) for downstream analysis. The dataset was stored as an.rds object and read into R using readRDS(). All analyses were performed using the default RNA assay, and cells were classified according to their assigned cell_classification identities as defined by PanKBase.

*SLC38A5* and *SLC38A4* expression data were extracted along with the corresponding cell type annotations using FetchData() and exported to CSV files for further inspection. Violin plots were generated using the raw data in GraphPad Prism.

### 2.5 Alanine stimulation and serum glucagon measurement

For alanine stimulation experiments, mice were fasted for 6 hours prior to testing. Baseline blood samples were collected via retro-orbital bleed under isoflurane anesthesia. Mice then received an intraperitoneal injection of L-alanine (Sigma, A7469) at a dose of 2 mg/g body weight, prepared at 100 mg/mL in sterile DPBS (Corning, MT21031CV). A second blood sample was collected at 15 minutes post-injection under isoflurane anesthesia.

Blood was collected into heparinized microcollection tubes (Fisher Scientific, 02-668-15) and transferred to 1.5 mL microcentrifuge tubes containing Halt Protease and Phosphatase Inhibitor (Thermo Fisher Scientific, 78442) at a 1:100 dilution. Samples were allowed to clot on ice for at least 30 minutes and centrifuged at 2,000 × g for 10 minutes at 4 °C. Clarified serum was transferred to low-binding microcentrifuge tubes (Thermo Fisher Scientific; 3449) and stored at −80 °C until analysis.

Serum glucagon concentrations were measured using the Mercodia Glucagon ELISA (10 µL format; catalog #10-1281-01) according to the manufacturer’s instructions. Absorbance was measured using a microplate reader (BioTek, Synergy H4).

### 2.6 Statistical analysis

All data are shown with error bars indicating standard error of the mean. Data within individual experiments were compared with ordinary two-way ANOVA using Tukey correction for multiple comparisons, unless otherwise designated in the figure legend. For the bar graph analysis, we excluded mice that did not respond to antibody treatment. These mice were defined as mice receiving antibody treatment that did not have elevated amino acid levels above the highest IgG mouse. No knockout mice were excluded. All mice are included in the correlation analyses to show the relationship between endpoints regardless of drug efficacy.

## 3 Results

### 3.1 SLC38A5 expression in α cells supports amino acid-induced α-cell proliferation

**Figure 1:**
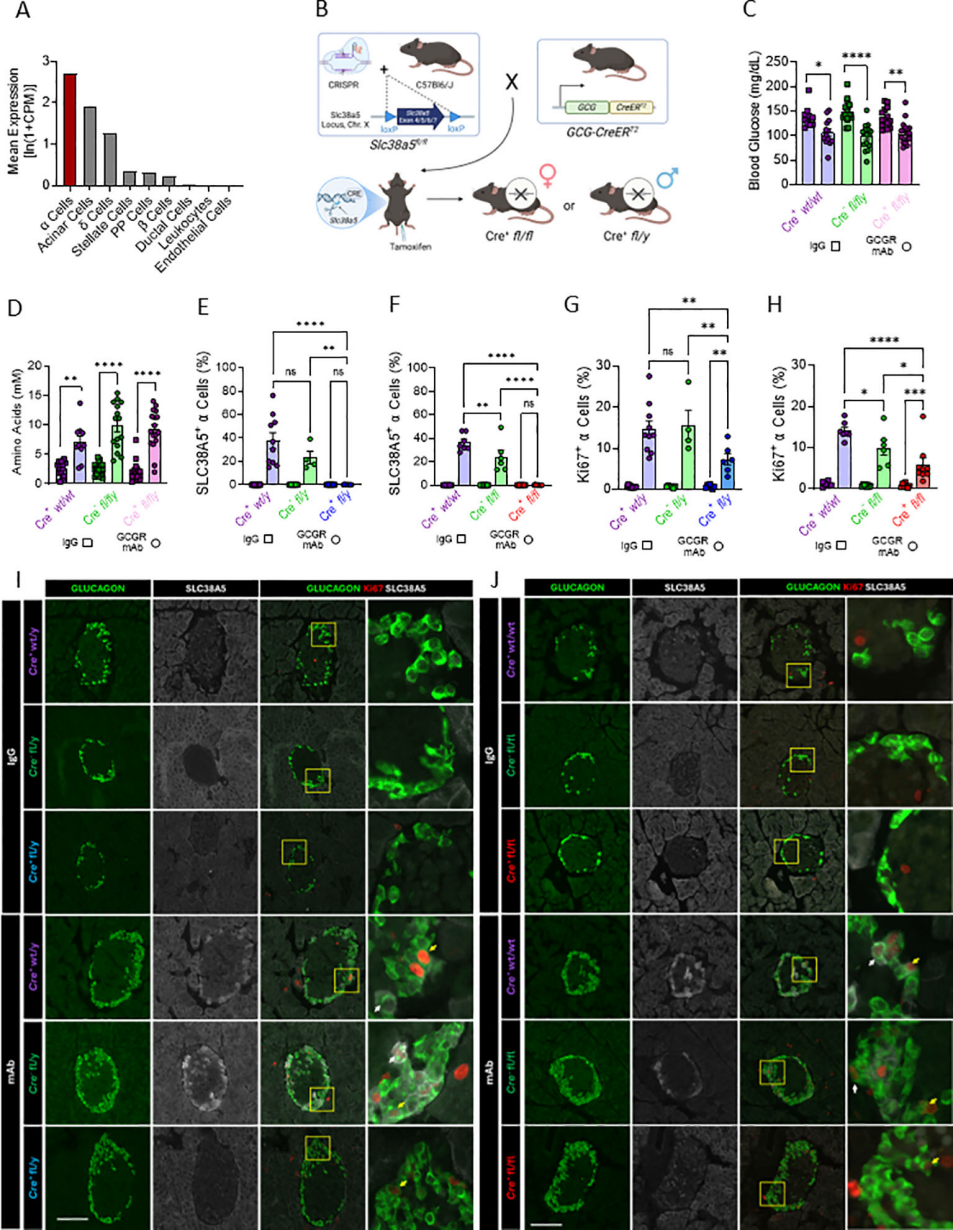
SLC38A5 expression in α cells supports amino acid-induced α-cell proliferation. **(A)** Relative *Slc38a5* expression in mouse pancreas cell types is shown. *Slc38a5* expression is highest in pancreatic α cells (red bar), followed closely by acinar cells and δ cells. **(B)** Schematic visualizing the generation of a new α-cell specific *Slc38a5* mouse model. Slc38a5 is found on the X chromosome and found from basepairs 8137390-8146418. The floxed alleles excised exons 4-7. **(C)** Random-fed blood glucose was measured in both male and female mice (fl/fly) following 2 weeks of GCGR monoclonal antibody (circles) or IgG (squares). Purple bars indicate Cre positive controls, green bars indicate Cre negative flox controls, and pink bars indicate the knockout mice. **(D)** Circulating amino acid levels were measured in both male and female mice (fl/fly) following 2 weeks of GCGR monoclonal antibody (circles) or IgG (squares). Purple bars indicate Cre positive controls, green bars indicate Cre negative flox controls, and pink bars indicate the knockout mice. **(E, F)** SLC38A5 protein levels were assessed by immunofluorescence after 2 weeks of GCGR monoclonal antibody or IgG control treatment in male **(E)** and female **(F)** mice. Purple bars indicate Cre positive controls, green bars indicate Cre negative flox controls, blue bars indicate male knockouts, and red bars indicate female knockouts. **(G, H)** Proliferation in α cells was measured by Ki67 staining and expressed as a percentage of total α cells in males **(G)** and females **(H)**. Purple bars indicate Cre positive controls, green bars indicate Cre negative flox controls, blue bars indicate male knockouts, and red bars indicate female knockouts. **(I, J)** Representative images of glucagon and Ki67 following 2 weeks of GCGR monoclonal antibody or IgG treatment in male **(I)** and female **(J)** mice. Glucagon is green. SLC38A5 is white. Ki67 is red. Scale Bar: 100uM, Insert Size: 25uM Yellow arrows indicate Ki67+Glucagon+ cells. White arrows indicate SLC38A5+Glucagon+ cells. *p<0.05, **p <0.01, ***p<0.001, ****p<0.0001.

Analysis of gene expression across pancreatic cell types revealed that *Slc38a5* is most highly expressed in acinar cells and pancreatic α cells compared with other pancreatic cell types, indicating a strong α cell–specific expression pattern (25) (**Figure 1A**). In humans, both *SLC38A5* and *SLC38A4*, a related system N sodium-coupled neutral amino acid transporter, are most highly expressed in α cells compared with other endocrine cell types (**Supplementary Figures 1A, B**) (26–39). These findings suggest that members of the system N transporter family play a central role in α-cell amino acid sensing and potentially in nutrient-regulated α-cell function.

Previous studies suggest that global loss of *slc38a5*/*Slc38a5* expression results in impaired amino acid induced-α cell proliferation in both zebrafish and mice (14, 23). Therefore, it is unclear if SLC38A5 expression changes in α cells drive increases in glucagon secretion or α cell proliferation. To address this, we generated *Slc38a5* floxed mice and crossed them to previously generated Gcg-Cre ERT2 mice to generate α cell-specific *Slc38a5* knockout mice (Cre^+^ *fl/fl*, Cre^+^ *fl/y*, or Cre^+^ *fl/fly*) (**Figure 1B**). To assess the regulation of circulating amino acids, mice were treated with a glucagon receptor monoclonal antibody (GCGR mAb), which robustly increased circulating amino acid levels. GCGR mAb treatment significantly decreased glycemia and increased amino acid levels across all genotypes (**Figures 1C, D**). There were no significant differences between genotypes in the IgG-treated mice (**Figure 1D**). Mice treated with GCGR mAb had 2–3 fold higher circulating amino acids than their IgG-treated littermates with no differences observed between genotypes (**Figure 1D**). Importantly, α cell-specific deletion of *Slc38a5* did not alter circulating amino acid concentrations in either males or females (**Figure 1D**), indicating that loss of SLC38A5 in α cells does not affect systemic amino acid elevation induced by GCGR blockade.

The gene encoding the neutral amino acid transporter SLC38A5 (*Slc38a5*) is located on the X chromosome (40), raising the possibility of sex-specific regulation of its expression (**Figure 1B**). In both male and female mice, α–cell–specific deletion of *Slc38a5* resulted in a ~99% reduction in SLC38A5 expression after tamoxifen injection, confirming efficient and comparable knockout across sexes. Notably, SLC38A5 expression was low under basal conditions and was strongly induced by ~35-fold only in the context of elevated circulating amino acids following GCGR antibody treatment (**Figures 1E, F**). In female floxed control mice, GCGR antibody treatment was associated with slightly lower SLC38A5 levels compared with *Cre*-negative controls, despite intact gene expression (**Figure 1F**).

Control mice exhibited robust α-cell proliferation following GCGR antibody treatment. Despite comparable elevations in circulating amino acids, amino acid–induced α-cell proliferation was markedly reduced in α-cell-specific *Slc38a5* knockout mice in both males and females (**Figures 1G–J**). These findings indicate that SLC38A5 expression within α cells is required for the proliferative response to elevated amino acids, rather than changes in systemic amino acid availability.

### 3.2 Amino acid-induced mTORC1 activation in α cells is independent of SLC38A5 expression

**Figure 2:**
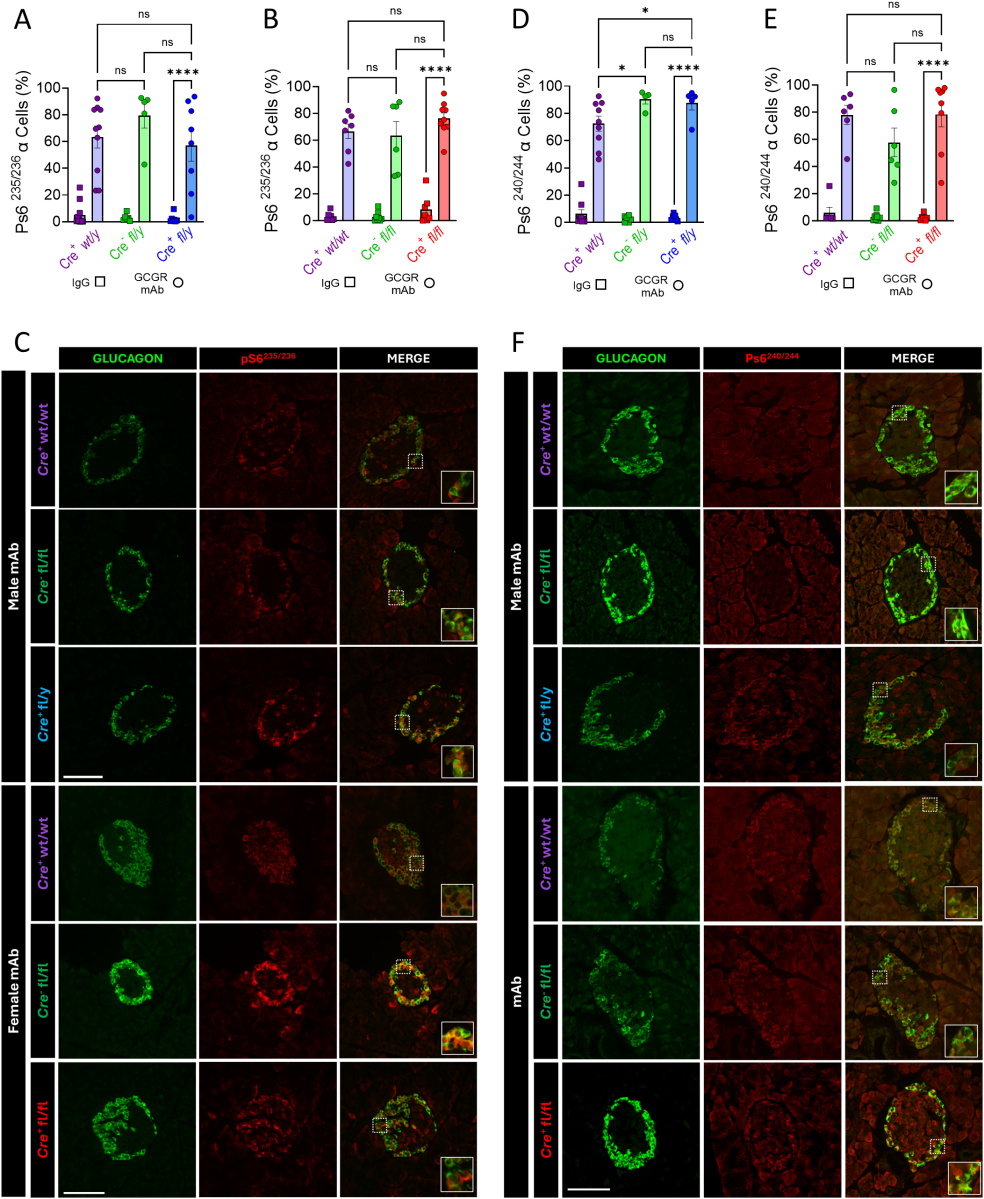
Amino acid-induced mTORC1 activation in α cells is independent of SLC38A5 expression. **(A)** Phosphorylation of S6 protein at Ser235/236 was assessed by immunofluorescence after 2 weeks of GCGR monoclonal antibody or IgG control treatment in male mice. **(B)** Phosphorylation of S6 protein at Ser235/236 was assessed by immunofluorescence after 2 weeks of GCGR monoclonal antibody or IgG control treatment in female mice. **(C)** Representative images of pS6 Ser235/236 (red) expression in male and female mice treated with GCGR mAb. Glucagon is green. **(D)** Phosphorylation of S6 protein at Ser240/244 was assessed by immunofluorescence after 2 weeks of GCGR monoclonal antibody or IgG control treatment in male mice. **(E)** Phosphorylation of S6 protein at Ser240/244 was assessed by immunofluorescence after 2 weeks of GCGR monoclonal antibody or IgG control treatment in female mice. **(F)** Representative images of pS6 Ser240/244 (red) expression in male and female mice treated with GCGR mAb. Glucagon is green. Scale Bar: 100uM, Inset Size: 10uM. *p<0.05, ****p<0.0001.

To determine whether α-cell-specific deletion of SLC38A5 affects mTORC1 signaling, we assessed phosphorylation of ribosomal protein S6 at Ser235/236, a commonly used readout of nutrient-responsive signaling (41). Immunofluorescence analysis revealed robust pS6 Ser235/236 staining in α cells from both control and knockout mice following two weeks of GCGR monoclonal antibody treatment (**Figures 2A–C**; **Supplementary Figures 2A, B**). Quantification indicated no significant differences in pS6 Ser235/236 levels between genotypes in either males or females regardless of amino acid levels, suggesting that α-cell SLC38A5 is not required for overall phosphorylation of S6 protein at this site (**Figures 2A, B**). These data indicate that while SLC38A5 is necessary for amino acid-induced α-cell proliferation, it does not appear to influence global pS6 Ser235/236 signaling under the conditions tested. Since phosphorylation of S6 at Ser235/236 can reflect inputs beyond canonical mTORC1 activity, we next examined Ser240/244 phosphorylation, which is more tightly associated with mTORC1-dependent signaling (42). Similarly, pS6 Ser240/244 expression in α cells was robustly increased following the 2-week GCGR mAb treatment (**Figures 2D–F**; **Supplementary Figures 2C, D**). Quantification revealed that there was no significant difference in pS6 Ser240/244 expression between the floxed controls and the knockout mice in both males or females (**Figures 2D, E**). While no significant differences in pS6 Ser240/244 expression were observed between Cre controls and other genotypes in females, male Cre controls exhibited significantly reduced pS6 Ser240/244 expression compared to both the floxed controls and the knockout mice (**Figures 2D, E**).These findings indicate that loss of SLC38A5 does not globally impair mTORC1 signaling but instead suggests that mTORC1 activation occurs independently of SLC38A5 expression.

### 3.3 Circulating amino acids positively correlate with SLC38A5, Ki67, and pS6

**Figure 3:**
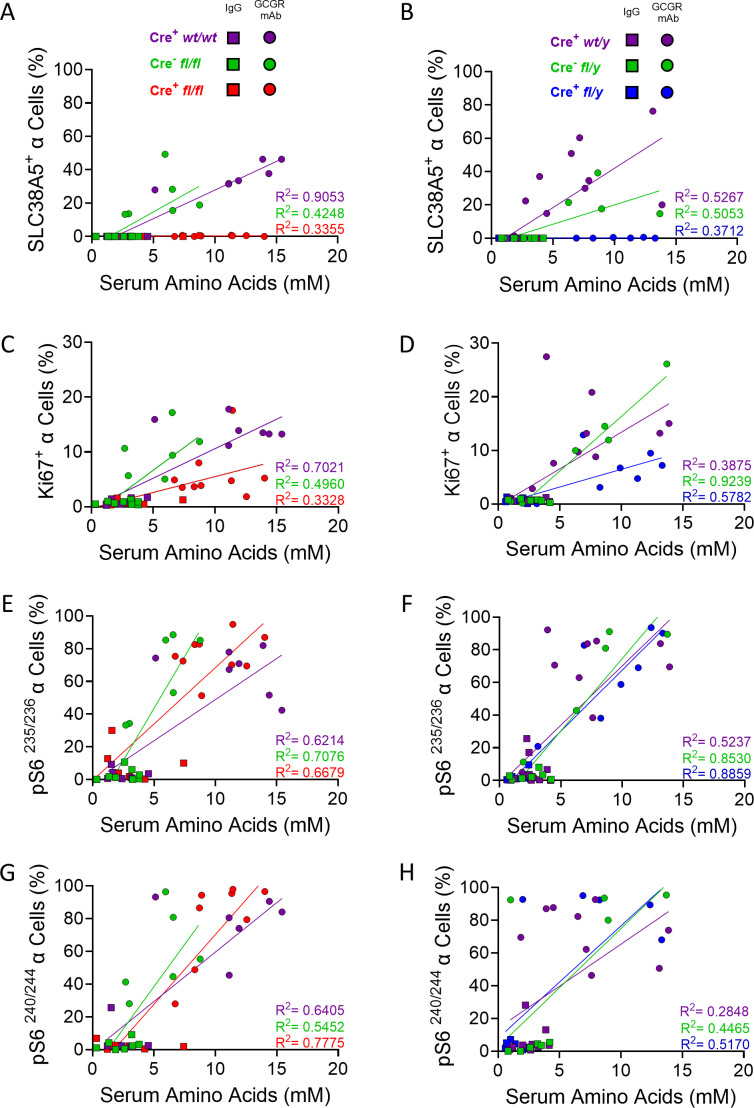
Circulating amino acids positively correlate with SLC38A5, Ki67, and pS6. **(A, B)** Correlation between circulating amino acid concentrations and the percentage of SLC38A5+ α cells in mice treated with either GCGR monoclonal antibody (circles) or IgG control (squares) males **(A)** and females **(B)**. **(C, D)** Correlation between circulating amino acids and α-cell proliferation, quantified as the percentage of Ki67+ α cells, in males **(C)** and females **(D)**. **(E, F)** Correlation between circulating amino acids and the percentage of pS6 (Ser235/236)+ α cells in male **(E)** and female **(F)** mice. **(G, H)** Correlation between circulating amino acids and the percentage of pS6 (Ser240/244)+ α cells in male **(E)** and female **(F)** mice. Correlations were assessed using Pearson’s correlation coefficient.

Circulating amino acid concentrations positively correlated with α cell-specific SLC38A5 protein expression in both male and female mice (**Figures 3A, B**). In female floxed control mice, this correlation exhibited a shallower slope compared with males, with a smaller increase in SLC38A5 per unit increase in amino acids, suggesting a modest sex difference in α-cell responsiveness (**Figure 3B**). In α-cell-specific SLC38A5 knockout mice, SLC38A5 protein levels remained minimal in both sexes, and no increase was observed despite elevated amino acid concentrations (**Figures 3A, B**). Amino acid levels also positively correlated with α-cell proliferation, as measured by the percentage of Ki67-positive α cells. This correlation was markedly blunted in knockout mice, indicating that SLC38A5 is required for the full proliferative response to amino acids (**Figures 3C, D**). A positive correlation between amino acid levels and pS6 (Ser235/236) expression was observed in α cells from both males and females. However, the loss of SLC38A5 did not affect this correlation, suggesting that amino acid-dependent phosphorylation at this site occurs independently of SLC38A5 (**Figures 3E, F**). A similar relationship was observed for pS6 Ser240/244, with amino acid levels positively correlating with α-cell pS6 Ser240/244 expression in both sexes, and this correlation was likewise unaffected by loss of SLC38A5 (**Figures 3G, H**). Together, these data indicate that SLC38A5 mediates the link between circulating amino acids and α-cell proliferation, while mTORC1 activation is SLC38A5-independent.

### 3.4 Loss of SLC38A5 in α cells impairs stimulated glucagon secretion in α cells

**Figure 4:**
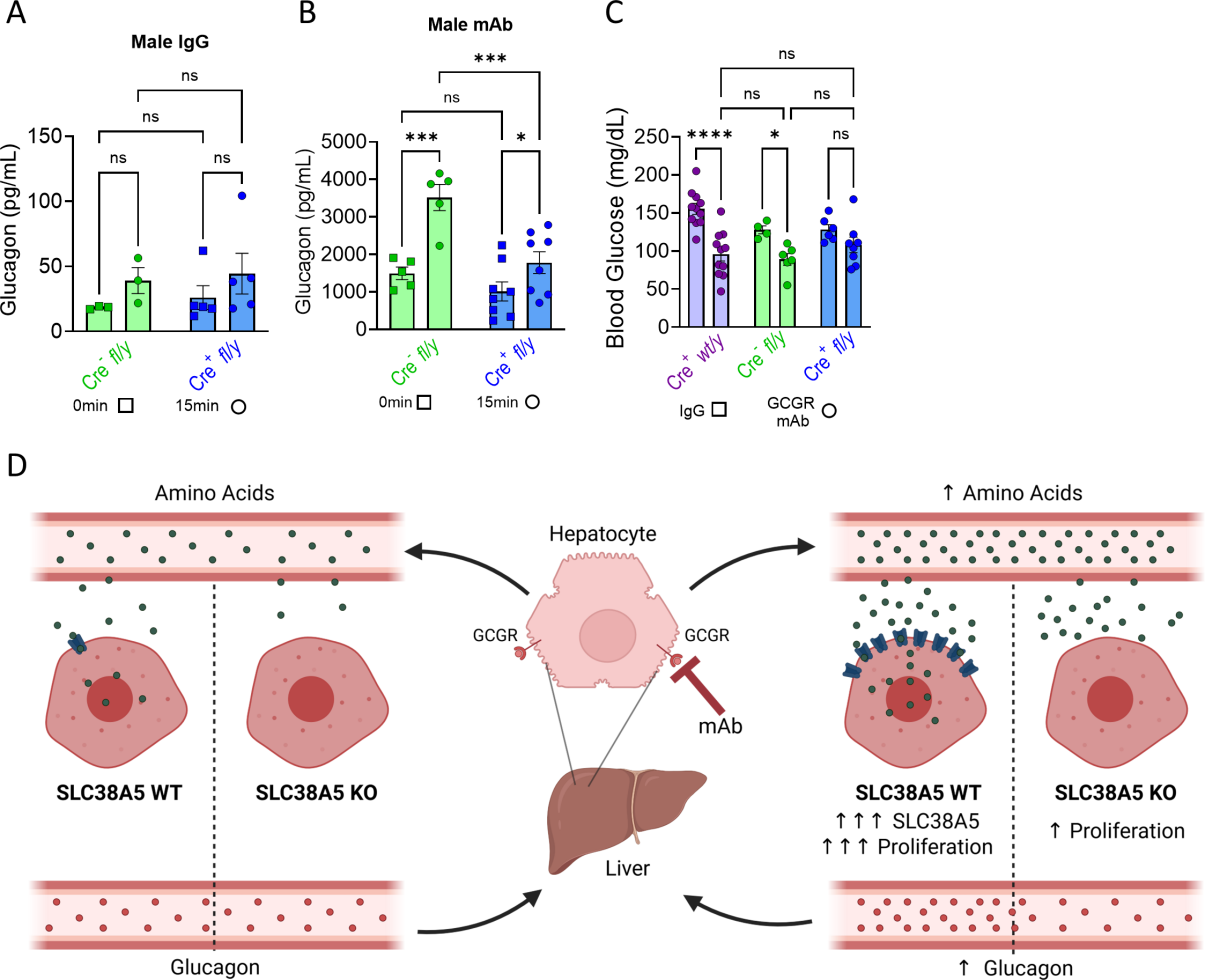
Proposed model of SLC38A5 regulation of α-cell proliferation. **(A)** Serum glucagon levels at baseline (0 min) and 15 min following amino acid stimulation in IgG-treated control male mice. **(B)** Serum glucagon levels at baseline (0 min) and 15 min following amino acid stimulation in GCGR monoclonal antibody-treated male mice. **(C)** Random-fed blood glucose was measured in males. **(D)** Schematic model illustrating the proposed role of SLC38A5 in α-cell amino acid sensing, proliferation, and glucagon secretion under conditions of elevated circulating amino acids. *p<0.05, ***p<0.001, ****p<0.0001.

To assess whether SLC38A5-dependent α-cell proliferation translates into functional changes in hormone secretion, we measured glucagon release following alanine stimulation. In IgG-treated males, serum glucagon rose modestly from baseline to 15 minutes after stimulation, although this increase did not reach statistical significance (**Figure 4A**). In contrast, GCGR mAb-treated control mice exhibited robust stimulated serum glucagon, whereas loss of SLC38A5 markedly blunted this response, despite comparable elevations in circulating amino acids (**Figure 4B**). These data suggest that when SLC38A5 expression is induced under GCGR mAb treatment, it increases the sensitivity of α cells to alanine. No significant differences in blood glucose were observed over the 2-week treatment period (**Figure 4C**), indicating that impaired glucagon response was not associated with altered systemic glycemia. Together, these findings support a model in which SLC38A5 in α cells is required to couple amino acid availability to appropriate glucagon secretory responses and proliferative adaptation (**Figure 4D**).

## 4 Discussion

Our study provides new insight into the role of the neutral amino acid transporter SLC38A5 in pancreatic α-cell biology. Using an α-cell-specific knockout model, we demonstrate that SLC38A5 is highly enriched in α cells in both mice and humans and is required for amino acid–induced α-cell proliferation in both males and females, establishing it as a critical mediator linking circulating amino acids to the adaptive expansion of α-cell mass. Deletion of SLC38A5 markedly blunted proliferation despite comparable elevations in circulating amino acids, indicating that the transporter functions cell-autonomously to regulate α-cell growth.

Interestingly, phosphorylation of S6 at Ser235/236, a commonly used readout of nutrient-sensitive signaling, was not affected by SLC38A5 deletion, even under conditions of elevated amino acids. This finding suggests that while SLC38A5 is necessary for amino acid-dependent α-cell proliferation, global signaling at this residue is largely SLC38A5-independent. Normally, SLC38A5 is restricted to the acinar cells postnatally (43). However, it is robustly expressed in α cells during fetal development (43). This coincides with a heavy reliance on amino acids during development and the robust expression of their transporters (44, 45).

Given that Ser235/236 can be phosphorylated by multiple kinases, including but not limited to mTORC1, our data indicate that SLC38A5 selectively modulates proliferative responses without broadly altering all nutrient-responsive signaling pathways. Similar results were found when investigating the phosphorylation of S6 at Ser240/244 in α cells following GCGR mAb treatment in mice lacking α-cell SLC38A5. Together, these findings align with our previous reports demonstrating the induction of SLC38A5 expression is sensitive to rapamycin indicating that mTOR activity upregulates SLC38A5 expression to potentiate amino acid–driven α-cell proliferation (**Figure 4D**) (14). Our findings also revealed subtle sex differences. Female floxed control mice exhibited a shallower slope in the correlation between amino acids and SLC38A5 expression, suggesting that α-cell responsiveness to amino acids may differ between sexes.

It is important to consider that many studies have identified mTORC1 as a central nutrient-sensing pathway regulating β-cell growth, proliferation, and function in response to metabolic cues (46, 47). However, a key consideration in reconciling these findings is that α and β cells exhibit fundamentally distinct nutrient sensing and intracellular metabolism despite sharing the same islet microenvironment (48, 49). β cells are classically highly glucose-responsive, coupling glucose metabolism to ATP generation and downstream mTORC1 activation to regulate growth and insulin secretion (45, 50–52), whereas α cells are comparatively less glucose-dependent and instead demonstrate heightened sensitivity to amino acid availability (15, 53–55). In particular, α cells are known to respond robustly to amino acid signaling, which can directly influence glucagon secretion and may engage alternative growth and signaling pathways independent of canonical mTORC1 activation (15). Thus, SLC38A5-mediated amino acid transport may preferentially activate α–cell–specific nutrient-sensing mechanisms or parallel proliferative pathways that are less dependent on mTORC1 than those operating in β cells. These intrinsic differences in substrate utilization and signaling hierarchy between α and β cells suggest that mTORC1 dependence may be cell-type–specific rather than universally required across islet endocrine populations.

Disruption of glucagon receptor signaling has been shown to induce marked α-cell adaptations, including single α-cell hypersecretion despite alterations in circulating glucagon levels (56, 57). Functionally, loss of SLC38A5 in mice treated with mAb had reduced glucagon response to amino acid stimulation, demonstrating that the transporter contributes not only to α-cell proliferation but also to nutrient-stimulated hormone output. However, reduced circulating glucagon following α cell-specific SLC38A5 knockout cannot be attributed solely to decreased α-cell mass. The reduction in stimulated glucagon occurred despite no significant changes in blood glucose, highlighting a specific defect in nutrient sensing rather than generalized α-cell dysfunction. While the observed phenotype is consistent with reduced α-cell abundance, the current experimental design does not directly resolve whether SLC38A5 also regulates per-cell glucagon secretory capacity. Given that amino acid transport through SLC38A5 can influence intracellular nutrient sensing pathways, SLC38A5 could contribute to the functional state of individual α-cells in addition to its role in proliferation. Therefore, future work will be required to determine whether SLC38A5 deletion in α cells alters single-cell glucagon secretion dynamics independently of changes in cell number.

It is important to note that our studies were performed in mice on a mixed genetic background, and that the Cre+ control mice were not littermates of floxed or knockout animals. These factors may contribute to subtle baseline differences, such as the reduced pS6 Ser240/244 observed in male Cre controls and should be considered when interpreting sex-dependent or genotype-specific effects. Notably, previous work using a global *Slc38a5* knockout model reported significant reductions in pS6 Ser240/244 in α cells (23), suggesting that systemic glutamine handling can influence mTORC1 signaling in α cells. Together, these observations indicate that while our α-cell-specific deletion demonstrates that SLC38A5 acts cell-autonomously to support proliferation, global nutrient transport may also modulate α-cell signaling *in vivo*.

Overall, this work establishes SLC38A5 as a central mediator of amino acid-induced α-cell adaptation. By facilitating amino acid uptake, SLC38A5 supports both proliferation and nutrient-stimulated glucagon secretion. However, its loss does not broadly impair mTORC1 signaling, as evidenced by preserved phosphorylation of S6 at Ser235/236 and Ser240/244. These findings improve our understanding of α-cell nutrient sensing and highlight SLC38A5 as a regulator for intracellular α-cell metabolism. Future studies will be needed to dissect the precise intracellular signaling pathways downstream of SLC38A5 that drive proliferation and glucagon release.

## Data Availability

The datasets presented in this study can be found in online repositories. The names of the repository/repositories and accession number(s) can be found in the article/**Supplementary Material**.

## Supplementary material

Supplementary Figure 1 **(A, B)** Analysis of *SLC38A5* **(A)** and SLC38A4 **(B)** expression through scRNA sequencing of human pancreatic endocrine cells. **(C, D)** Circulating amino acid levels were measured in both male **(C)** and female **(D)** mice following 2 weeks of GCGR monoclonal antibody (circles) or IgG (squares).

Supplementary Figure 2 **(A)** Representative images of pS6 Ser235/236 expression in male mice treated with IgG. **(B)** Representative images of pS6 Ser235/236 expression in female mice treated with IgG. **(C)** Representative images of pS6 Ser240/244 expression in male mice treated with IgG. **(D)** Representative images of pS6 Ser240/244 expression in female mice treated with IgG.
