# Supplementary figures and images for: α-cell SLC38A5 supports amino acid-induced α-cell proliferation and glucagon secretion
Source: Front Endocrinol (Lausanne). 2026 Jun 15;17:1830329. doi: 10.3389/fendo.2026.1830329 (PMC13310714; doi:10.3389/fendo.2026.1830329)

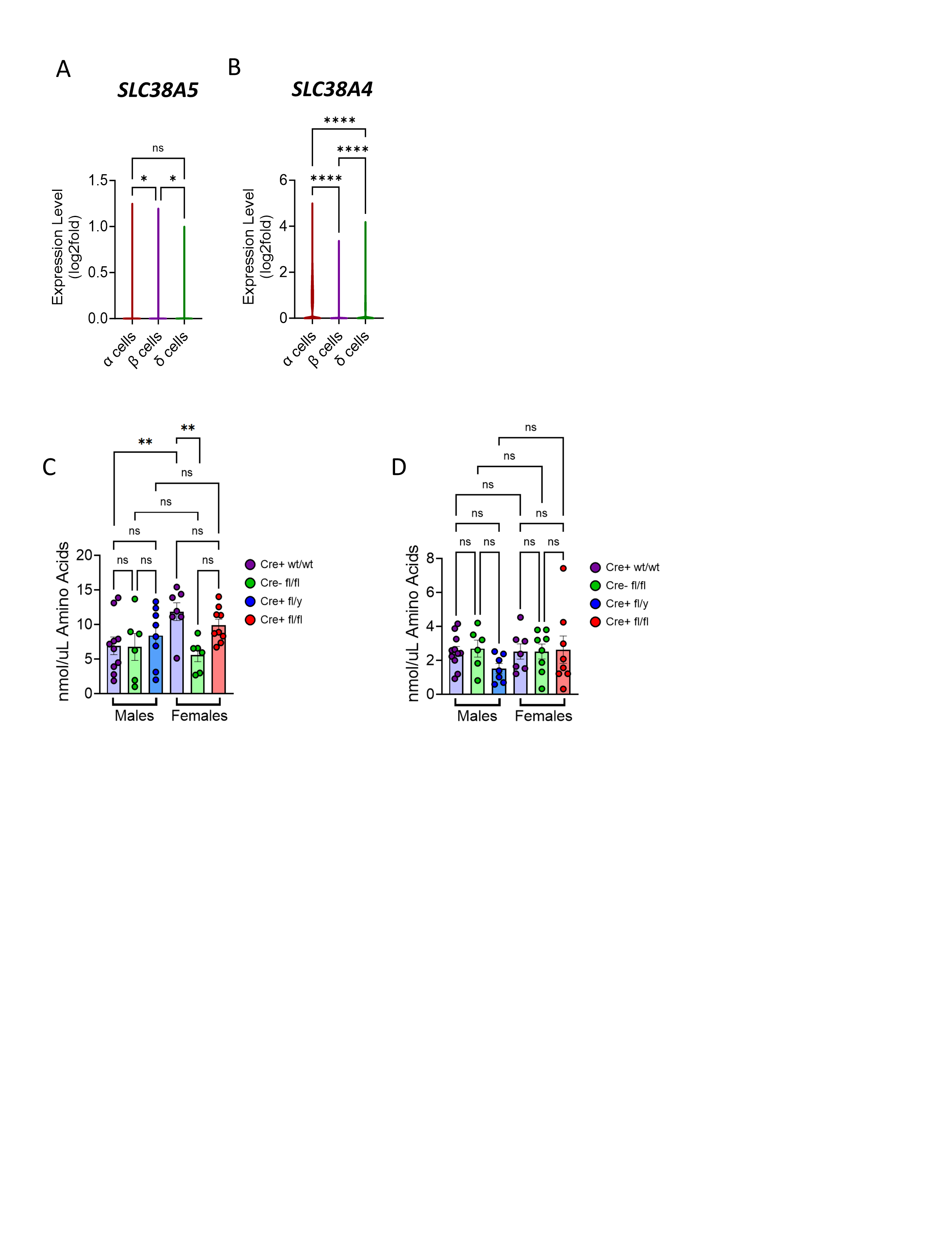

Supplement: Supplementary Figure 1 — (A, B) Analysis of SLC38A5 (A) and SLC38A4 (B) expression through scRNA sequencing of human pancreatic endocrine cells. (C, D) Circulating amino acid levels were measured in both male (C) and female (D) mice following 2 weeks of GCGR monoclonal antibody (circles) or IgG (squares). [file Image1.tif]

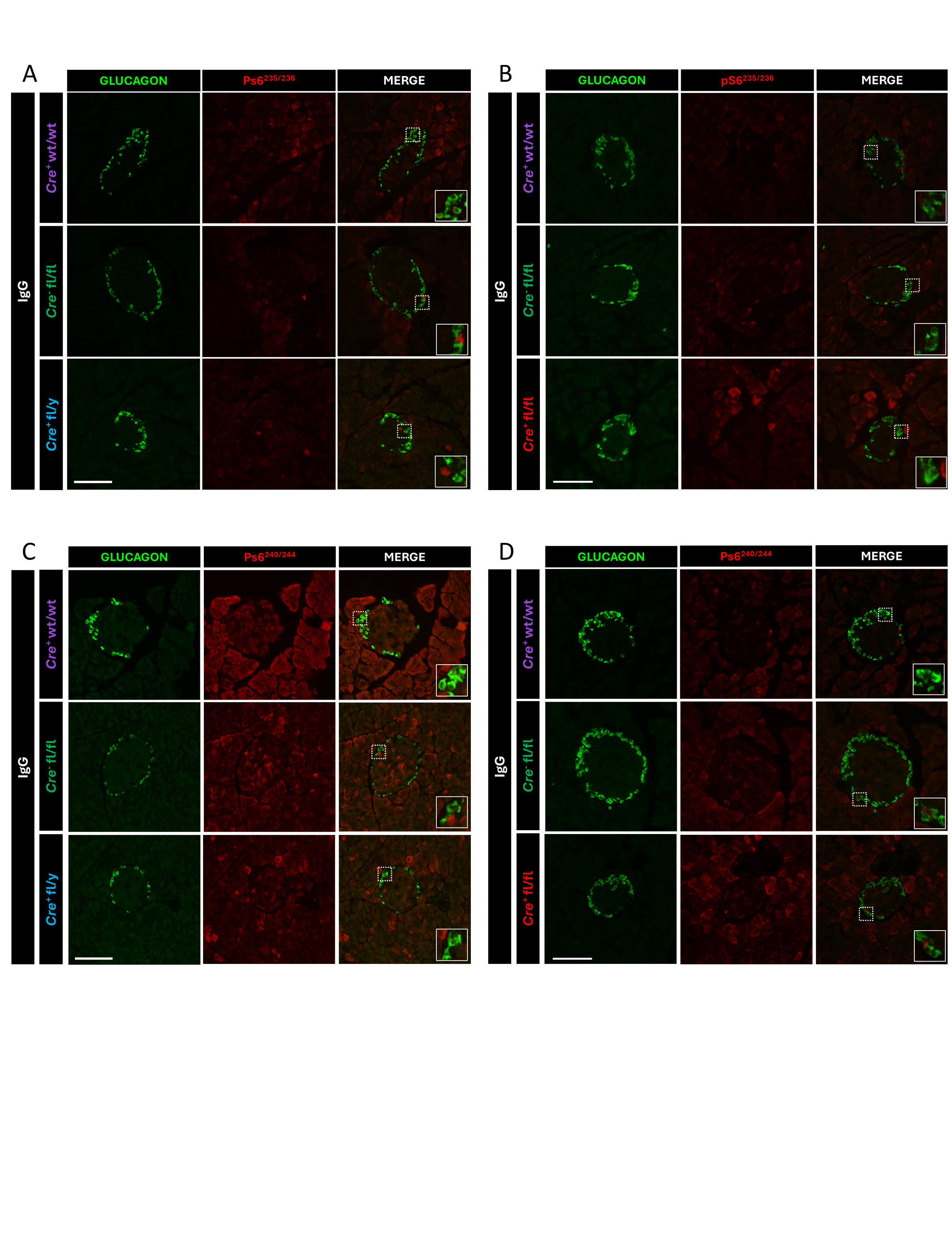

Supplement: Supplementary Figure 2 — (A) Representative images of pS6 Ser235/236 expression in male mice treated with IgG. (B) Representative images of pS6 Ser235/236 expression in female mice treated with IgG. (C) Representative images of pS6 Ser240/244 expression in male mice treated with IgG. (D) Representative images of pS6 Ser240/244 expression in female mice treated with IgG. [file Image2.tif]
